# Morphological and Molecular Characterization of *Talanema eshtiaghii* sp. n. (Dorylaimida, Qudsianematidae) from Iran

**DOI:** 10.2478/jofnem-2023-0022

**Published:** 2023-06-05

**Authors:** Nasir Vazifeh, Gholamreza Niknam, Habibeh Jabbari, Reyes Peña-Santiago

**Affiliations:** Department of Plant Protection, Faculty of Agriculture, University of Tabriz, Tabriz, Iran; Department of Plant Protection, Faculty of Agriculture, University of Maragheh, Maragheh, Iran; Departamento de Biología Animal, Biología Vegetal y Ecología, Universidad de Jaén, Campus ‘Las Lagunillas’ s/n, Edificio B3, 23071-Jaén, Spain

**Keywords:** Bayesian inference, D2–D3 rDNA, phylogeny, taxonomy

## Abstract

A new species of the genus *Talanema*, recovered from the northwest of Iran, was described based on morphological, morphometric, and molecular data. *Talanema* eshtiaghii sp. n. was characterized by its 1.45–1.68 mm long body, lip region offset by constriction and 13–15 μm wide, odontostyle 15–18 μm long, double guiding ring, neck 312–362 μm long, pharyngeal expansion occupying 41–43% of the total neck length, uterus tripartite, and 111–189 μm long or 2.1–3.2 body diameters, vulva transverse (V = 55–58), tail similar in both sexes, conical with a dorsal concavity (30–44 μm, c = 33–56, c’ = 1.0–1.6), spicules 49–56 μm long, and 14–18 shortly spaced ventromedian supplements in front of the level of the anterior end of spicules, with distinct hiatus. It was compared to four closely similar species, with emphasis on the most relevant traits to distinguish them. Molecular phylogenetic studies using partial sequence of the 28S rDNA (D2–D3 segment) revealed that the new species forms a clade with other currently sequenced representatives of *Talanema*, tentatively supporting the monophyly of this genus.

Andrássy (1991) proposed the genus *Talanema* to accommodate four species transferred from *Labronema* Thorne, 1939. Other species were later on added by Vinciguerra and Clausi (1994), Shaheen and Ahmad (2005), and Andrássy (2011). More recently, the genus was matter of two monographic contributions (Imran et al., 2021; Jabbari et al., 2021), including the first molecular study of one of its representatives using 18S, D2–D3 28S rDNA data. Besides, Peña-Santiago et al. (2022) have described its thirteenth species from the Iberian Peninsula.

Jabbari et al. (2021) studied one previously undescribed and three known species from Iran, where the genus apparently is well represented and diverse. Recently, another Iranian population was collected during a nematological survey conducted in the country. Its study confirmed that it belongs to an unknown species, which is described in this contribution.

## Material and Methods

### Sampling, morphological and morphometric study

Several soil samples were collected from the Sufiyan district, East Azarbaijan province, northwestern Iran. The modified method of Brown and Boag (1988) was used to extract nematodes from soil samples. Nematodes were transferred to anhydrous glycerine according to De Grisse's (1969) method and mounted on glass slides. Morphological observations were made and morphometrics were taken using an Olympus BX41 microscope with a drawing tube device. Micrographs were taken using a DP50 digital camera attached to the same microscope, powered with differential interference contrast (DIC). Drawings were made using the CorelDRAW® software version 12.

### DNA extraction, PCR and sequencing

A single nematode specimen of the new species was picked out and transferred to a small drop of distilled water or worm lysis buffer (WLB) and crushed by a sterilized scalpel. The suspension was transferred to a microtube containing 25.65 μl ddH_2_O, 2.85 μl 10X PCR buffer, and 1.5 μl proteinase K (600 μg/ml) (Promega, Benelux, the Netherlands). The microtube was incubated at 65°C (1 h), then at 95°C (15 min). The resulting DNA extract was stored at −20°C until used as a template for polymerase chain reaction (PCR). For DNA amplification, 1 μl of the extracted DNA was transferred to a microtube containing: 0.75 μl of each primer, 12.5 μl *Taq* DNA Polymerase 2× Master Mix RED, 2Mm MgCl_2_ (Amplicon-Denmark) and ddH2O to a final volume of 25 μl. The thermal cycler was programmed as follows: denaturation at 94°C for 2 min, followed by 35 cycles of denaturation at 94°C for 30 sec, annealing at 55°C for 45 sec, and extension at 72°C for 3 min. A final extension was performed at 72°C for 10 min (Archidona-Yuste et al., 2016). Primers for 28S rDNA D2–D3 amplification were D2A (5′-ACAAGTACCGTGAGGGAAAGT-3′) as forward primer and D3B (5′ TGCGAAGGAACCAGCTACTA-3′) (Nunn, 1992) as reverse primer. The PCR product was sequenced in both directions using the same primers used in PCR with an Applied Biosystems® 3730/3730xl DNA Analyzer in South Korea. The newly generated sequence of the studied species was deposited in GenBank database (accession number OP870361).

### Phylogenetic analyses

The recently obtained LSU rDNA sequence was edited/trimmed and compared with other dorylaimid sequences available in the GenBank database using the BLAST homology search program of the National Centre for Biotechnology Information (NCBI). The selected DNA sequences were aligned using the Muscle software implemented in MEGA6 (Tamura et al., 2013). MrModeltest 2.3 (Nylander, 2004) was used to select the base substitution model supported by the Akaike criterion in conjunction with PAUP* v4.0b10 (Swofford, 2003). Bayesian analysis (BI) was performed using MrBayes 3.1.2 (Ronquist and Huelsenbeck, 2003) running the chains for 10 million generations. After discarding burn-in samples, the remaining samples were retained for further analyses. Posterior probabilities (PP) are given on appropriate clades. The tree was visualized using FigTree v1.4.3 and was digitally drawn in CorelDRAW software version 12.

## Results

### Systematics

**Figure 1:**
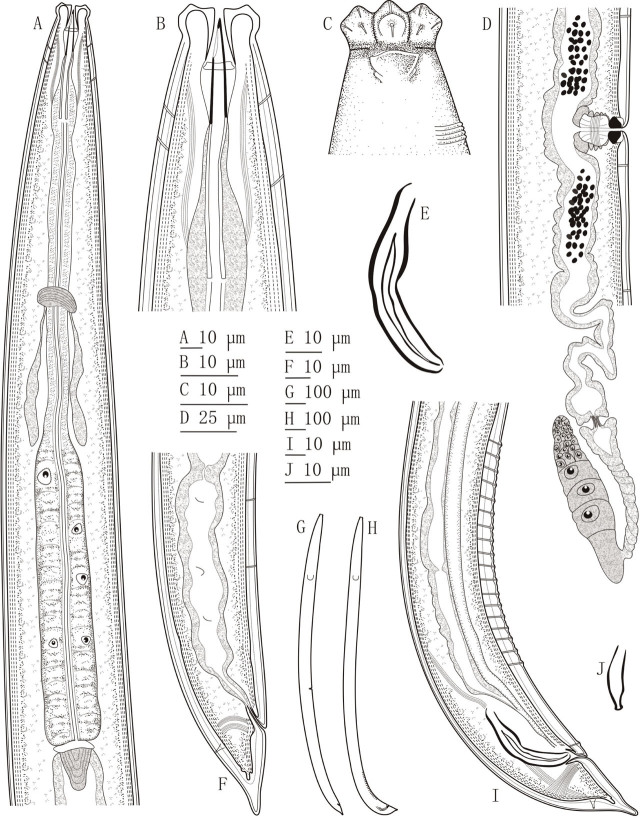
*Talanema eshtiaghii* sp. n. (A) neck region; (B) anterior region in lateral median view; (C) anterior region in lateral surface view; (D) female, posterior genital branch; (E): spicules; (F) female, caudal region; (G) female, entire; (H) male, entire; (I) male, caudal region; (J) lateral guiding piece.

**Figure 2:**
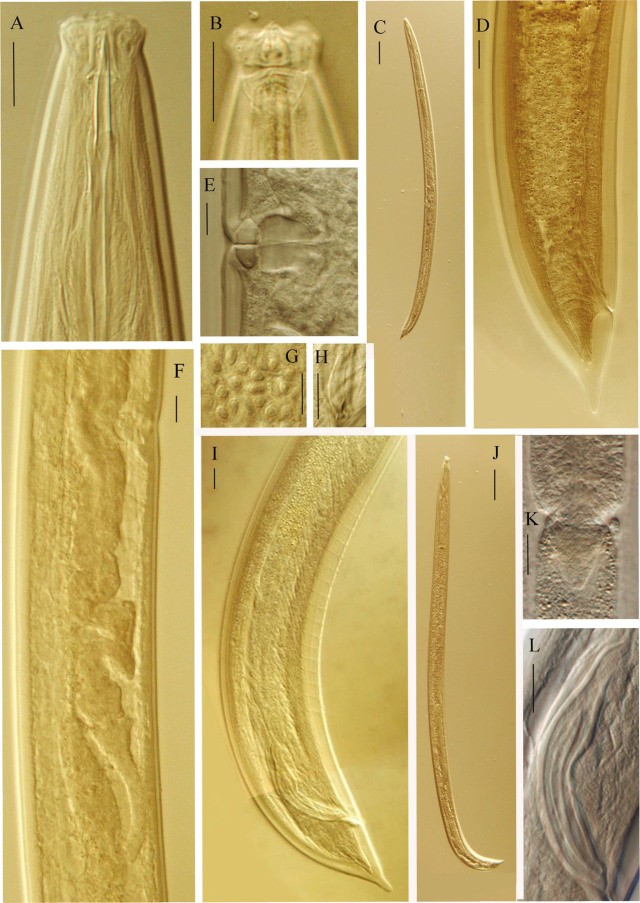
*Talanema eshtiaghii* sp. n. (A) anterior region in lateral median view; (B) anterior region in lateral surface view; (C) female, entire; (D) female, caudal region; (E) vagina region: (F) female, posterior genital branch; (G) sperm cells; (H) lateral guiding piece; (I) male, caudal region; (J) male, entire; (K) pharyngo-intestinal junction; (L) spicules. (Scale bars: A, B, D–I, K and L = 10 μm, C and J = 100 μm).

*Talanema eshtiaghii* sp. n. (Figs. 1, 2)

### Morphometrics

See Table 1.

**Table 1.** Morphometrics of *Talanema eshtiaghii* sp. n. from Iran. Measurements in μm except L in mm, and in the form: average ± sd (range).

**Locality Province**	**City of Sufiyan, Roodghat area, Zeinabad village** **East Azarbaijan**		

	**Holotype**	**Paratypes**	

**n**	♀	5♀♀	3♂♂
Character			
L	1.50	1.56 ± 0.08 (1.45–1.68)	1.49 ± 0.04 (1.45–1.53)
a	32	29.2 ± 2.1 (27–32)	27.6 ± 2.1 (27–30)
b	4.3	4.5 ± 0.2 (4.3–4.9)	4.5 ± 0.1 (4.4–4.6)
c	44	42.1 ± 7.7 (33–56)	39.7 ± 3.6 (37–44)
c’	1.2	1.3 ± 0.1 (1.0–1.6)	1.1 ± 0.1 (1.0–1.3)
V	55	56 ± 1.2 (55–58)	-
Lip region diameter	13	13.7 ± 0.1 (13–15)	13.4 ± 0.1 (13–14)
Odontostyle length-dorsal side	18	16.4 ± 1.3 (15–18)	17.3 ± 0.5 (17–18)
Odontophore length	24	25.1 ± 1.7 (24–27)	26.1 ± 0.7 (25–27)
Neck length	344	349 ± 10 (341–362)	322 ± 11 (312–337)
Pharyngeal expansion length	144	141.0 ± 7.6 (131–152)	133.0 ± 2.1 (124–138)
Body diameter at neck base	45	51.1 ± 4.3 (45–56)	52.0 ± 3.3 (49–56)
mid-body	47	52.3 ± 2.8 (47–59)	53.4 ± 3.5 (50–58)
anus/cloaca	27	28.3 ± 1.2 (27–30)	32.3 ± 1.5 (31–34)
Prerectum length	61	67 ± 10 (56–81)	73.4 ± 8.3 (59–97)
Rectum/cloaca length	34	34.2 ± 2.8 (30–38)	59.0 ± 4.1 (58–66)
Tail length	34	38.7 ± 3.6 (30–44)	36.1 ± 3.8 (33–40)
Spicules length	-	-	52.2 ± 3.5 (49–56)
Ventromedian supplements	-	-	(14–18)

### Description

#### Adult

Moderately slender to slender (a = 27–32) nematodes of medium size, 1.45–1.68 mm long. Body cylindrical, tapering towards both ends, but more so posteriorly, as the tail is conical in both sexes. Upon fixation, habitus slightly curved ventrad, to an open C shape. Cuticle almost smooth under light microscopy, two-layered, its total thickness 1.5–2.0 μm at anterior region, 2.5–4.0 μm in mid-body, and 4.0–4.5 μm on dorsal side of tail. Lateral chord 10.5–14.5 μm or 21–24% of maximum body diameter. Lip region offset by a weak but distinct constriction, 2.5–3.2 times as wide as high and less than one-third (24–32%) of body diameter at neck base; lips moderately separate and slightly angular, and perioral liplets might be present, labial and cephalic papillae visibly protruding. Amphidial fovea cup-shaped, its aperture 7.5–9.0 μm long or up to two-thirds (57–67%) of lip region diameter. Cheilostom a truncate, thick-walled cone. Odontostyle robust, hardly but appreciably shorter at its ventral side, 6.4–7.4 times as long as wide, longer (1.1–1.2 times) than lip region diameter, its aperture 5.5–7.0 μm or one-third to two-fifths (32–41%) of the total length. Guiding ring double, fixed ring situated at 9–11 μm or 0.7 times the lip region diameter from the anterior end. Odontophore rod-like, 1.3–1.6 times longer than odontostyle. Pharynx entirely muscular, gradually enlarging into the basal expansion that is 5.9–6.9 times longer than wide, 2.6–3.2 times longer than body diameter at neck base, and occupies less than one-half (39–43%) of the total neck length; gland nuclei located as follows: DO=59–60, DN=62–68, S_1_N_1_=70–75, S_1_N_2_=77–79, S_2_N=85–87. Nerve ring located at 126–146 μm distance from anterior end or 34–43% of the total neck length. Pharyngo-intestinal junction consisting of a short and rounded conoid cardia enveloped by intestinal tissue, all together forming a longer conoid, 14–19 × 12–15 μm structure, bulging into the intestinal lumen.

### Female

Genital system diovarian, with equally developed branches, the anterior branch 260–322 μm long or 16–20% of body length, the posterior one 233–322 μm or 16–20% of body length. Ovaries moderately developed, often reaching and surpassing the sphincter level, 60–74 μm the anterior and 60–79 μm the posterior, with oocytes first arranged in two or more rows, then in only one row. Oviduct joining subterminally the ovary, 62–89 μm or 1.1–1.5 times the body diameter long, consisting of a slender distal region made of prismatic cells and an often well-developed proximal *pars dilatata* with lumen inside. Sphincter present between oviduct and uterus. Uterus tripartite, usually convoluted, 111–189 μm long or 2.1–3.2 body diameters, consisting of a distal, almost spherical, *pars dilatata* with visible lumen, a long and slender intermediate section without visible lumen and somewhat refractive lining, and a proximal dilated region with wide lumen. Uterine egg not observed. Vagina extending inwards 23–25 μm to 38–44% of body diameter: *pars proximalis* 15.6–17 × 10–11.2 μm, with almost straight walls surrounded by weakly developed musculature, *pars refringens* consisting of (lateral view) two close together, often mostly trapezoidal, sclerotized pieces measuring 4.5–5.5 × 3–3.5 μm and with a combined width of 8–9 μm, and *pars distalis* 3–4 μm long. Vulva a slightly post-equatorial, transverse slit. Prerectum 2.0–2.7, rectum 1.1–1.3 anal body diameters long. Caudal region conical with finely rounded terminus, ventrally almost straight, dorsally first convex, then with a more or less (usually well) distinct concavity, giving a digitate aspect to the tail; inner core reaching 52–59% of tail length, leaving an appreciable hyaline portion; caudal pores one pair, at the middle of tail, one subdorsal, another lateral.

#### Male

Genital system diorchic, with opposite testes. In addition to the ad-cloacal pair, situated at 7.5 μm from the cloacal aperture, there is a series of 14–18, almost contiguous or shortly spaced, 6.0–7.5 μm apart, ventromedian supplements, the most posterior of them situated at 49–51 μm from the ad-cloacal pair, appreciably in front of the anterior end of spicules, then with a distinct hiatus. Spicules dorylaimid, 4.9–5.5 times as long as wide, 1.5–1.8 times the body diameter at level of the cloacal aperture: head 10.0–16.5 μm long or 18–32% of spicule length, 2.2–2.4 times longer than wide, and its dorsal side much longer and more curved than the ventral one; median piece comparatively slender, occupying 25–28% of spicule maximum width; posterior tip 3.5 μm wide; ventral hump located at 18–22 μm or 36–42% of spicule length from its anterior end; curvature 134º. Lateral guiding piece 13–14 μm long, coarse, 3.4–4.4 times as long as wide, conspicuously tapering at its posterior third. Prerectum 1.8–3.7, cloaca 1.8–1.9 body diameters long. Caudal region basically similar to that of female, but more straight or even slightly curved ventrad at the end.

#### Molecular characterization

Sequencing the D2–D3 region of the 28S rDNA resulted in one sequence 751 bp (OP870361) long. The BLAST homology search showed it has an 89.09% identity with *Talanema baqrii* (Khan et al., 1989; Imran et al., 2021) (MT645228), 91% identity with *T. ibericum* (Peña-Santiago et al., 2022) (OP793646) and 94% identity with *Talanema* sp. (OP870362).

#### Diagnosis

The new species is characterized by its 1.45–1.68 mm long body, lip region offset by constriction and 13–15 μm wide, odontostyle 15–18 μm long, guiding ring double, neck 312–362 μm long, pharyngeal expansion occupying 41–43% of the total neck length, uterus tripartite and 111–189 μm long or 2.1–3.2 body diameters, vulva transverse (V = 55–58), tail similar in both sexes, conical with a dorsal concavity (30–44 μm, c = 33–56, c’ = 1.0–1.6), spicules 49–56 μm long, and 14–18 shortly spaced ventromedian supplements in front of the level of the anterior end of spicules, with distinct hiatus.

#### Relationships

Morphologically and morphometrically, the new species resembles three *Talanema* species showing no sexual dimorphism of tail shape, namely, *T. ibarakiense* (Khan and Araki, 2002; Andrássy, 2011), *T. pararapax* (Ahmad and Jairajpuri, 1982; Andrássy, 1991) and *T. sphinctum* (Mohilal and Dhanachand, 2001; Imran et al., 2021), but it can be distinguished from these in its shorter odontostyle (15–18 versus 20 μm long or more). Besides, it differs from *T. ibarakiense* in its larger general size (body length 1.45–1.68 versus 1.0–1.2 mm), more posterior vulva (V = 55–58 versus 50–52), longer tail (30–44 versus 21–24 μm, c’ = 1.0–1.6 versus 0.8–0.9), and less (14–18 versus 21–22) ventromedian supplements with distinct (versus without) hiatus. From *T. pararapa*x in its shorter spicules (49–56 versus 57–63 μm) and different arrangement of ventromedian supplements (ending in front of versus at level of the anterior end of spicules). It can be separated from the type population of *T. sphinctum* in its more posterior vulva (V = 55–58 versus V = 52–55), higher number of ventromedian supplements (14–18 versus 8) with very different arrangement (shortly versus widely spaced, with distinct versus no hiatus). It is also similar to *T. saccatum* (Jabbari et al., 2021), an Iranian monosexual species, from which the new species can be separated in its more slender body (a = 27–32 versus a = 22–26), somewhat shorter odontostyle (15–18 versus 18–20 μm), and slightly longer female tail (30–44 versus 24–31 μm, c’ = 1.0–1.6 versus c’ = 0.8–1.0) with no (versus abundant) saccate bodies. Finally, the new species also resembles *Labronema digiturum* (Vinciguerra, 1984), a taxon with a questionable belonging to *Labronema*, but it can be distinguished from this in its much more slender body (a = 27–32 versus a = 16–20), shorter odontostyle (15–18 versus 19–20 μm), tripartite (versus apparently simple) uterus, absence (versus presence) of cuticular irregularities (wrinkles) at both sides of vulva, and shorter spicules (49–56 versus 67 μm).

**Figure 3:**
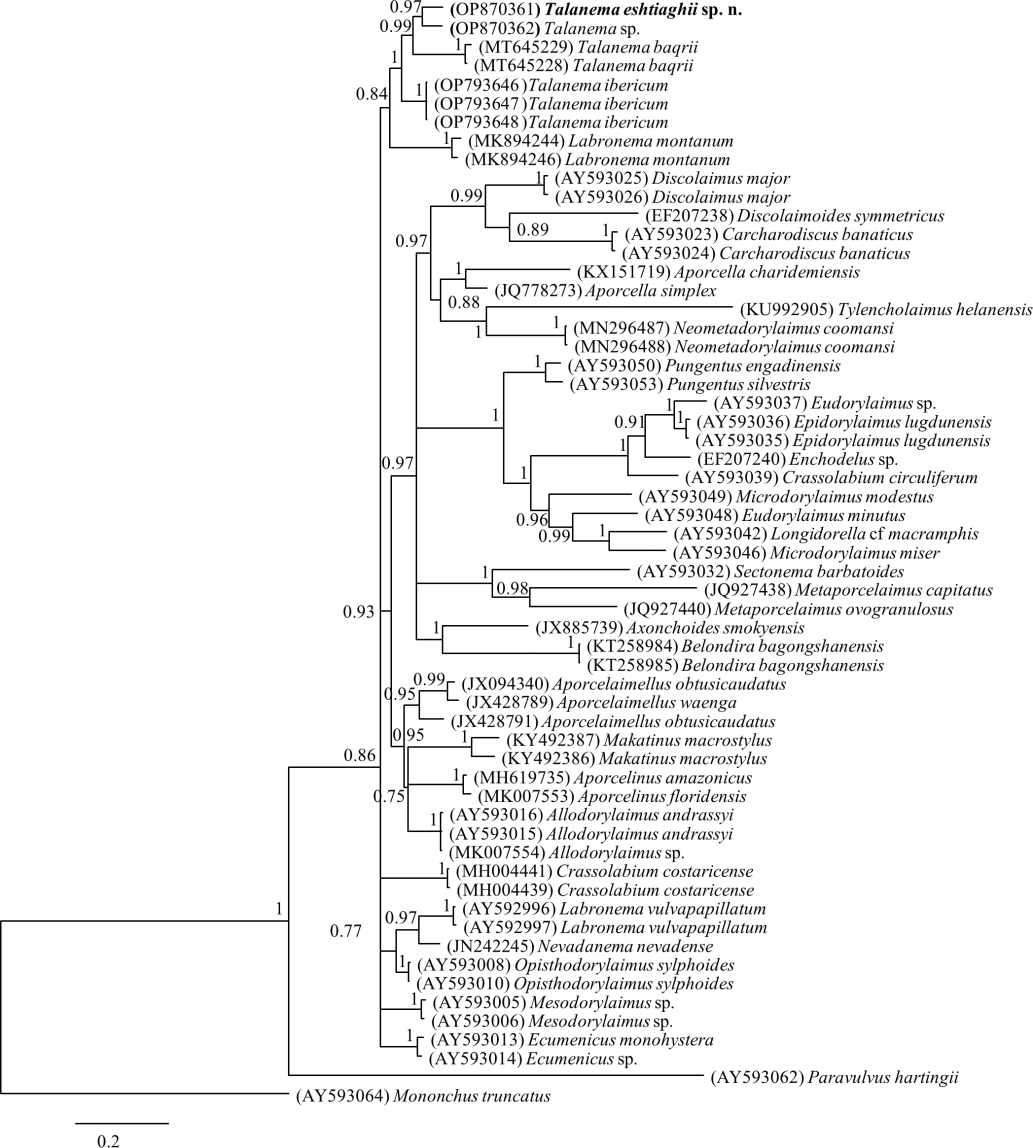
Bayesian 50% majority rule consensus tree of *Talanema eshtiaghii* sp. n. as inferred from D2–D3 expansion segments of 28S rRNA gene sequence under the GTR+I+G model. Bayesian posterior probabilities are given for each clade. Newly obtained sequence is indicated by bold letters.

The evolutionary relationships of the new species, as derived from the molecular analyses, are presented in the tree of Figure 3. The 28S rDNA sequence of *T. eshtiaghii* sp. n. formed a maximally supported clade (1.00 Bayesian posterior probability) with other *Talanema* representatives, namely *T. baqrii, T. ibericum* and *Talanema* sp. This means that, at present, *Talanema* is a monophyletic taxon. Nevertheless, its external relationships remain unsatisfactorily resolved. Thus, on the one hand, it forms part of a larger clade also including two sequences of *Labronema montanum* (Peña-Santiago and Abolafia, 2019) as a sister group, but with comparatively low support (0.84 BPP). On the other hand, this (*Talanema* + *Labronema*) clade is only one out of six Dorylaimina subclades, whose evolutionary relationships are neither well resolved.

#### Type locality and habitat

Northwestern Iran, East Azarbaijan province, Sufiyan district, Roodghat area, Zeinabad village (38°17′46″N, 46°07′37″E, elevation 1528 m a.s.l.), where the new species was collected in soil from the rhizosphere of common wheat (*Triticum aestivum* L.).

#### Type material

Female holotype, three female paratypes and two male paratypes deposited with Collection of Nematology Laboratory, University of Tabriz, Iran. One female paratype and one male paratype deposited in the Nematode Collection of the University of Jaén, Spain.

The LSID for this publication is urn:lsid:zoobank.org:act:250EA066-7CC1-48DD-94C5-4ADD6490E015

#### Etymology

The new species is named in honor of Dr. Hassan Eshtiaghi, the late Nematologist in the Department of Plant Protection, University of Tehran, Tehran, Iran.
